# Effect of Silk Fibroin on the Mechanical and Transport Properties of Agarose Hydrogels

**DOI:** 10.3390/gels10100611

**Published:** 2024-09-24

**Authors:** Veronika Richterová, Miloslav Pekař

**Affiliations:** Institute of Physical and Applied Chemistry, Faculty of Chemistry, Brno University of Technology, Purkynova 464/118, 612 00 Brno, Czech Republic; pekar@fch.vut.cz

**Keywords:** fibroin, agarose, hydrogel, rheology, mesh size, diffusion

## Abstract

In this work, the effect of incorporating silk fibroin, a fibrous biocompatible protein, into physically cross-linked agarose hydrogels was investigated as a simple model study to examine how supramolecular fibrous structures influence the properties of the hydrogels. The rheological and transport properties were studied. Fibroin did not change the general viscoelastic properties of the investigated hydrogels but changed the viscoelastic moduli values and also the mesh size, as calculated from rheometry data. Fibroin influenced the mechanical properties depending on its concentration: at lower concentrations, it increased the mesh size, while at higher concentrations, it acted as a filler, decreasing the mesh size. Similarly, the storage and loss moduli were affected, either increasing or decreasing based on the fibroin concentration. The fibroin effect on the diffusion of two dyes differing in their charge was the result of a combination of structural effects, responsible also for changes in the rheological properties, and a result of electrostatic interactions between the charged groups. For positively charged methylene blue, low fibroin concentrations accelerated diffusion, while higher concentrations slowed it by filling network vacancies. In contrast, for negatively charged eosin-B, fibroin strongly impeded diffusion at all concentrations due to electrostatic repulsion, leading to its accumulation at the hydrogel interface. The findings of this work may contribute to an understanding of the behavior of the extracellular matrix or soft tissues as well as to the development of the tailored design of hydrogel materials.

## 1. Introduction

Hydrogels are widely used materials in biomedical and other applications [1]. These three-dimensional materials based on hydrophilic polymers can absorb large amounts of water (or other biological fluids) while exhibiting unique properties such as biocompatibility, biodegradability, and viscoelasticity [2,3]. The variety of polymer types allows for the creation of hydrogels with unique and specific properties suitable for the desired applications, such as porosity or mechanical strength [4]. Since the introduction of hydrogel contact lenses [5], the application range of hydrogels has grown greatly. Nowadays, hydrogels are used in various areas, especially thanks to their properties, in tissue engineering and other medical applications. They are used in hygiene products, can serve as carrier systems for drug distribution, are used for wound healing and in regenerative medicine, where they serve as substitutes for damaged tissues, are used for the separation of biomolecules and cells, and they are suitable materials for biosensors or for use as substitutes for extracellular matrix [6,7,8,9,10].

Hydrogels are also used as artificial environments for cells, where they replace the cells’ native extracellular matrix [11,12,13]. However, the bioenvironment is not just a simple gel but has the character of a hydrocolloid that contains many different molecules that co-create the biological, chemical, and mechanical properties of the extracellular matrix. Through the interplay of these properties, the extracellular matrix forms a ‘home environment’ for the cells that allows their vital functions [14,15]. The natural intercellular matrix also includes various fibers such as collagen or elastin, which naturally also contribute to a suitable cellular environment. These fibrous molecular structures play a key role as structuring elements that influence, among other things, the mechanical properties of the extracellular matrix [16,17,18]. 

Many of these materials are created by combining different types of hydrogels, both natural and synthetic or combinations thereof, which ensures that the material has the most suitable properties [19,20,21]. Natural hydrogels often have properties similar to the extracellular matrix [22], and at the same time, materials of natural origin are often made up of various proteins or components of the matrix, such as collagen or hyaluronic acid [23,24]. Since they are derived from natural sources, they are both biocompatible and bioactive, with a very low immunological response [25,26]. The materials used mainly include polysaccharides (alginate, agarose, cellulose, chitosan, gellan gum, or hyaluronic acid) [27,28,29,30,31], but also various proteins (collagen and fibrin) [10,32] and composite materials (hyaluronic acid combined with polylysine or methylated cellulose) [24,33]. The disadvantage of using natural materials is the limited improvement of their mechanical and biochemical properties, as their modification may reduce biocompatibility. To improve their properties, these materials can be suitably combined [34]. 

One suitable material for creating an artificial environment is agarose, a natural and non-toxic linear polysaccharide made up of repeating units of agarobiose, which is obtained from the agar found in some species of seaweed [35]. Due to its properties, such as high water retention capacity, low immune response due to its inert structure, and controllable nutrient permeation, it is a suitable material for many medical applications. It finds use, sometimes independently, but more often in combination with other materials, in the controlled distribution of drugs and cell encapsulation; it can also serve as a wound cover, as a bioink, or in the formation of eye corneas, skin, and cartilage, and as a part of agar in the food industry [28,36,37]. Agarose is soluble in hot water, where its molecules are in the form of a random coil. The agarose chains cool into helical bundles primarily held together by hydrogen bonds but also by hydrophobic interactions. No additional cross-linking agents are needed for the formation of the hydrogel, which arises from non-covalent interactions to create supramolecular hydrogels [38,39]. However, agarose has some limitations, such as lower mechanical properties (in comparison to natural ECM) and thermal sensitivity, and it is not biodegradable in the human body [40,41,42].

Silk fibroin is a fibrous protein that is, thus, one of the materials widely used for forming hydrogel matrices and in tissue engineering despite not being a component of the native extracellular matrix. It is a versatile biocompatible material with slow degradation and is suitable for biomedical applications thanks to its extraordinary properties, such as low immunogenicity and remarkable mechanical properties [43,44,45]. The toughness of silk fibers is greater than that of synthetic materials, including Kevlar, as well as natural materials, such as collagen and polylactic acid [46]. Silk fibroin was selected as a representative of fibrous proteins, which is readily available and has already been applied in tissue engineering studies (bone, cartilage, soft tissues, cornea, blood vessels, and skin). Various diseases are modeled on it (breast cancer and polycystic kidney disease), and it is used for implants (fibroin sponges filling defects in bones and joints) [23,47,48].

This study aimed at a basic investigation of the effects of a fibrous structure on the rheological and diffusional properties of hydrogels. Macromolecular fibers are one of the essential components of the extracellular matrix, which is, in principle, a hydrogel environment. These fibers form supramolecular assemblies within the hydrogel matrix, and the resulting hydrogel can be considered a special type of supramolecular hydrogel, modeling the basic features of natural supramolecular hydrogel structures. Agarose, as the hydrogel-forming constituent, and silk fibroin, as the fibrous component, were used to create a primitive model of structures found either in the extracellular matrix or in engineered materials for use in regenerative medicine and cell culturing. Most studies are focused on specific materials in specific applications, including the agarose and fibroin we are using in our experiments [36,49]. However, the aim here is not to create a specific hybrid hydrogel but rather to present a comprehensive set of results that helps to understand how the presence of fibroin influences the mechanical and transport properties of agarose hydrogels. This approach highlights how the fibrous component affects the amorphous one, a concept inspired by the different compositions of the ECM. Of course, real materials like the extracellular matrix are of a much more complex composition, and this work should, therefore, be viewed as a contribution to the call for studies [50] on the influence of isolated components (of extracellular matrix) on transport and mechanical properties. The selected system was composed of a polysaccharide matrix imbued with protein fibrous chains and can also be viewed as an example of a semi-interpenetrated network.

## 2. Results and Discussion

Agarose was chosen on the basis of prior experience (both that of our team and many others) as a physically crosslinked hydrogel matrix suitable for investigating changes in hydrogel properties resulting from the incorporation of silk fibroin. Although silk fibroin can also form hydrogels [51], both chemically and physically cross-linked, none of the techniques that lead to the cross-linking of silk fibroin were used in our sample preparations, and in all samples, silk fibroin remained in the form of “free fibers.” Among the possibilities for the formation of silk fibroin hydrogel is self-assembly, in which β-sheet conformations are formed due to the thermodynamic instability of the silk fibroin solution. From preliminary experiments, it was found that self-assembly at laboratory temperature occurs within hours to days; however, the gelation of the agarose hydrogel occurs within a few minutes when cooled to laboratory temperature. Thus, it can be concluded that samples prepared in this way do not contain silk fibroin in the form of a hydrogel.

### 2.1. Characterization of Extracted Silk Fibroin

**Figure 1:**
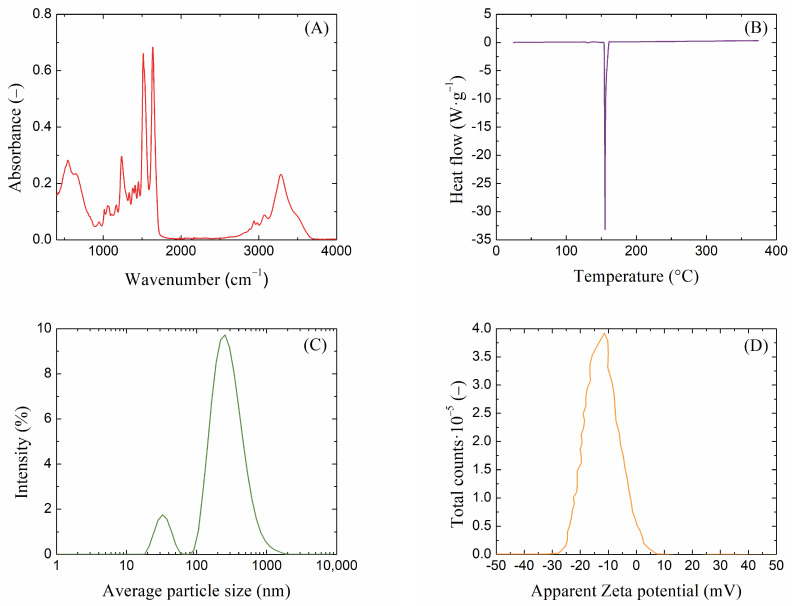
(**A**) FT-IR spectra, (**B**) DSC thermogram, (**C**) DLS size distribution, and (**D**) Zeta potential of extracted silk fibroin solution.

To properly handle macromolecular fibers such as silk fibroin, it is necessary to characterize these materials [52]. Infrared spectroscopy (FT-IR) is an excellent technique for obtaining important information about the structure and composition of a material based on characteristic absorption bands. The FT-IR of proteins encompasses various vibrations associated with different functional groups; the FT-IR spectrum of extracted silk fibroin is shown in Figure 1A. The absorption peaks around 1640, 1520, and 1240 cm^−1^ corresponded to the peptide backbone of amide I, the N–H bending of amide II, and the C–N stretching of amide III, respectively. The molecular conformation of silk fibroin is characterized by β-sheet absorption peaks around 1630, 1530, and 1240 cm^−1^, random coil conformation absorption peaks at 1650 or 1645, 1550, and 1230 cm^−1^, and an α-helix absorption peak at around 1655 cm^−1^ [53,54]. Since the silk fibroin solution is added to the hot agarose solution (80 °C), another convenient method that provides information on the thermal stability of the samples and, in the case of proteins, characterizes their thermal denaturation, is differential scanning calorimetry (DSC). From the DSC thermogram shown in Figure 1B, it can be observed that the isolated silk fibroin exhibited relatively good thermal stability. A very significant change occurred only at a temperature of 155 °C, which is the temperature at which fibroin breaks down into smaller peptides and amino acids [55,56]. This process causes the silk fibroin to lose strength and structural integrity but at a higher temperature than that used in the preparation of agarose gels with fibroin. To obtain information on the particle size of the extracted fibroin solution and to determine the charge, the method of dynamic light scattering (DLS) was used. In a diluted (2 wt. %) silk fibroin solution, there were particles with a size of approximately 250 nm (Figure 1C). Smaller particles (30 nm) that were found were likely to be fibers oriented in a different direction. When determining the ζ-potential of the fibroin solution, a slightly negative value of −12 mV was found (Figure 1D).

### 2.2. Rheological Characterization of Agarose/Silk Fibroin Hydrogels

Hydrogel rheology is an important tool for understanding the relationship between the chemical structure of hydrogels and their behavior at the macroscopic level. This knowledge is key to understanding the effects of components added to hydrogels, such as the fibroin in our case. To determine the viscoelastic properties of the resulting hydrogels, oscillatory rheological experiments were performed. These experiments were performed mainly to determine how the addition of silk fibroin affects the resulting viscoelastic properties of agarose hydrogels.

**Figure 2:**
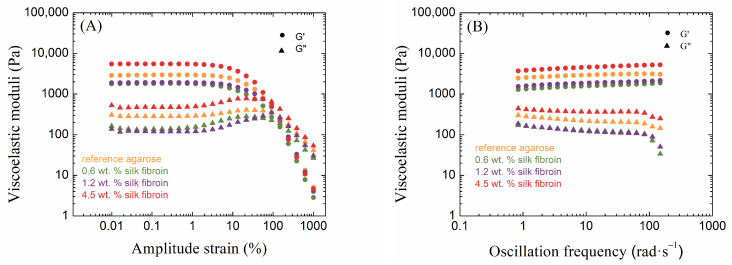
Amplitude sweep (**A**) and frequency sweep (**B**) tests of 0.5 wt. % agarose hydrogels (reference and with each addition of silk fibroin). Circles ● represent storage moduli G′ and triangles ▲ represent loss moduli G″.

Amplitude sweep tests provide information about the linear viscoelastic region (LVER), which is one of the most important characteristics of hydrogel rheology. The LVER is the range of amplitudes of deformations in which the storage and loss moduli are constant and where the hydrogel can resist the applied oscillatory strain. In addition to indicating the strength of non-covalent hydrogel nodes, the determination of the LVER is also key for performing frequency tests, which should be performed in this region. Other important information from amplitude tests includes the values of the viscoelastic moduli and the cross-over points of these moduli. The values of the viscoelastic moduli reflect the density of the resulting hydrogel network, which often indicates greater mechanical strength on the part of the hydrogel. When the values of these moduli equalize at the cross-over point, there is a change in the behavior of the material and a transition from solid-like to liquid-like behavior (in the case when G′ is initially greater than G″, i.e., when the elastic response prevails). The results from the oscillatory rheology measurements of 0.5 wt. % agarose with all silk fibroin additions are shown in the following Figure 2 (the remaining results are shown in Supplementary Material Figures S1 and S2).

Agarose hydrogels are ideal materials for studying mechanical properties from a rheological point of view. It is well known that as the concentration of agarose increases, the resulting hydrogels become mechanically stronger, which is also evident in oscillatory rheology measurements. As the concentration of agarose increases, the values of both viscoelastic moduli G′ and G″ increase, corresponding to the crosslinking density of the resulting hydrogel network. It is also possible to observe the influence on the LVER ends. The LVER length decreased with an increasing concentration of agarose. Thus, the region in which the sample is resistant to mechanical stress was shortened. The crossover point also shifts to lower strain amplitudes with increasing agarose concentrations; hence, with the denser crosslinking of the agarose hydrogel, irreversible deformation of the sample occurs earlier by changing from solid-like to liquid-like behavior. Corresponding to the crosslinking density and the values of the viscoelastic moduli, the more concentrated the agarose hydrogel is, the smaller the mesh size of the hydrogel.

The results from oscillatory rheology show different effects of fibroin on agarose hydrogels, relating to the relative concentrations of the components. Significant changes occurred in the linear viscoelastic region, where, in the case of low concentrations of agarose (0.5 and 1.0 wt. %), its length decreased due to the addition of fibroin when compared to the pure agarose (Table S1 in the Supplementary Materials); this length was greatest for the addition of fibroin at a medium concentration (1.2 wt. %). In contrast, the samples with a high concentration of agarose (2.0 wt. %) showed the opposite behavior. The addition of fibroin demonstrated the shortest LVER at its medium concentration (1.2 wt. %), its length shorter than in the case of pure agarose. The LVER was longer for the other two fibroin concentrations (0.6 and 4.5 wt. %) than for pure agarose.

**Figure 3:**
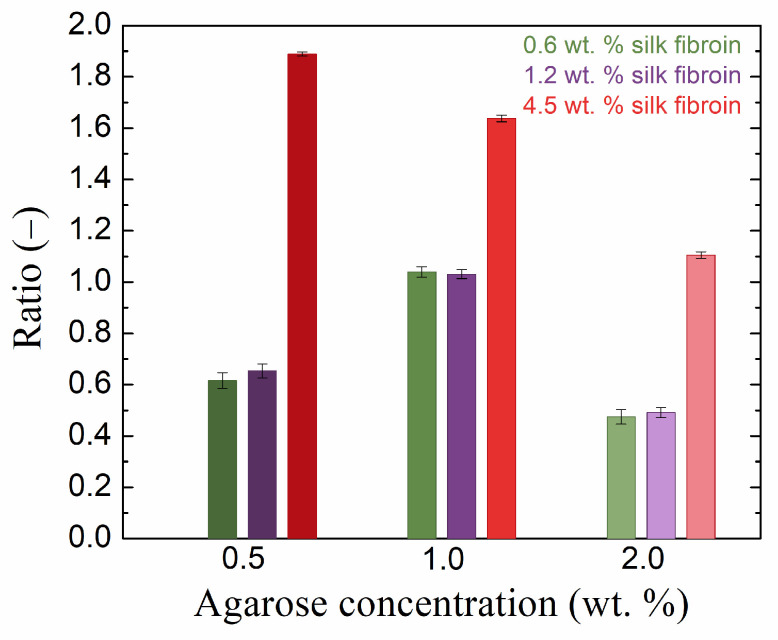
Ratio of the LVER viscoelastic modulus G′ of pure agarose gel to that of the gel with the addition of silk fibroin at various concentrations.

The storage modulus was significantly higher than the loss modulus for all samples in the LVER (Table S1 in Supplementary Materials), indicating dominant elastic behavior on the part of all the prepared hydrogels. The effect of fibroin addition on both moduli was significant and dependent on both the agarose and fibroin concentrations; an example of the elastic (storage) modulus is given in Figure 3. At the lowest concentration of agarose (0.5 wt. %), the addition of fibroin first decreased the values of both viscoelastic moduli, while at the highest concentration (4.5 wt. %), both moduli exceeded the values for pure agarose hydrogel significantly. A similar situation was found for the highest concentration of agarose (2.0 wt. %). In the case of the medium concentration of agarose (1.0 wt. %), the elastic modulus was always higher for the fibroin-added hydrogels, and the same results were found for the loss modulus, except for the sample with a fibroin concentration of 1. 2 wt. %. Figure 3 shows that the samples with the medium concentration of agarose responded differently to the addition of fibroin in comparison to the other two agarose concentrations used.

Changes due to the addition of fibroin to agarose hydrogels also occur at the cross-over points, i.e., changes in the corresponding common value of the moduli and in the value of the strain (Table S1 in Supplementary Materials). It should be noted that although cross-over occurs beyond the LVER, i.e., in a region of certain destructive changes, the cross-over points were reproducible in replicated amplitude sweep measurements. For pure agarose, the cross-over moduli value increased, and the strain decreased with increasing agarose concentrations. The smallest effect of fibroin addition was observed for the medium agarose concentration (1.0 wt. %). For the other two concentrations, the addition of fibroin at 0.6 and 1.2 wt. % decreased the cross-over moduli value and increased the corresponding strain, while at the highest concentration (4.5 wt. %), the moduli increased and the strain remained more or less unchanged.

The results of the frequency sweep measurements confirmed the solid-like behavior of all samples, i.e., the independence or very weak dependence of the moduli on frequency [57]. The effect of silk fibroin on moduli values was identical to its effect on moduli in the LVER.

**Figure 4:**
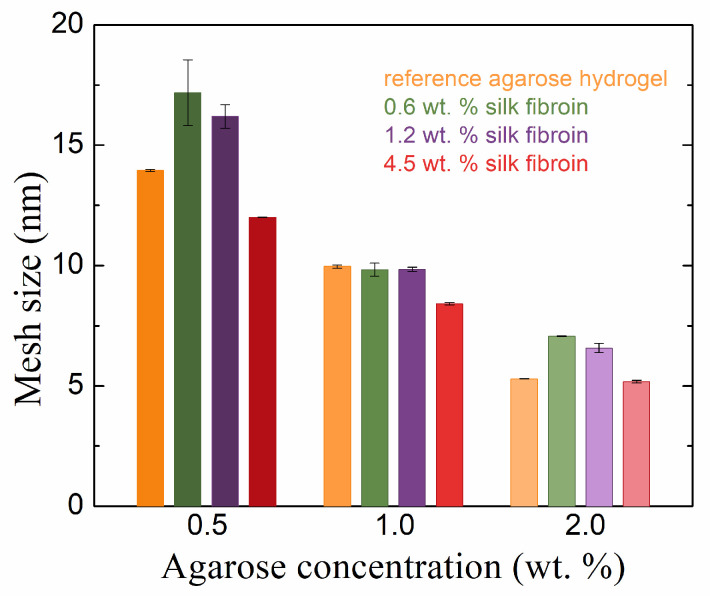
Rheologically calculated mesh sizes of agarose hydrogels with and without the addition of silk fibroin in different concentrations.

The addition of fibroin to agarose hydrogels also changed the mesh sizes in the hydrogel networks (Figure 4). Again, the samples had an agarose concentration of 1.0 wt. % behaved differently from the other two concentrations (cf., for example, Figure 3 and Figure 4). In the latter, with the addition of fibroin at 0.6 and 1.2 wt. %, the mesh size increased (relative to pure agarose), which was the result of the presence of fibroin inside the forming agarose network, hindering the physical crosslinking of the agarose chains. In the case of fibroin at the highest concentration (4.5 wt. %), the mesh size was smaller (0.5 wt. % agarose) or comparable (2.0 wt. % agarose) to that of pure agarose. The decreased mesh size indicates the filling of the original agarose network with fibroin fibers and/or the incorporation of fibroin into the agarose network. A decreased mesh size was also observed for increased agarose concentrations in our hydrogels.

The rheological results demonstrate that the addition of fibroin had significant effects on the mechanical properties of agarose hydrogels, with the impact depending on the relative concentrations of both agarose and fibroin. Importantly, while fibroin did not alter the overall viscoelastic behavior, such as the shapes of strain amplitude or frequency curves, it did affect the specific values of storage and loss moduli and the mesh size. At lower concentrations, fibroin seemed to interfere with the formation of the agarose network, increasing the mesh size. In contrast, at higher concentrations, fibroin acted as a filler, decreasing the mesh size by occupying vacancies within the hydrogel. The effects on the cross-over points of moduli, indicative of transitions from solid-like to liquid-like behavior, and on the strain at which this transition occurs, were also found to be fibroin concentration-dependent. The results suggest that the ability of fibroin to interact with agarose chains, both physically and electrostatically, can be fine-tuned by adjusting the fibroin concentration. These findings are essential for understanding how fibrous components like fibroin can modulate the structural integrity and mechanical behavior of hydrogels, which may have implications for their application in areas such as tissue engineering and controlled release systems.

### 2.3. Diffusivity of Dyes in Agarose–Silk Fibroin Hydrogels

**Figure 5:**
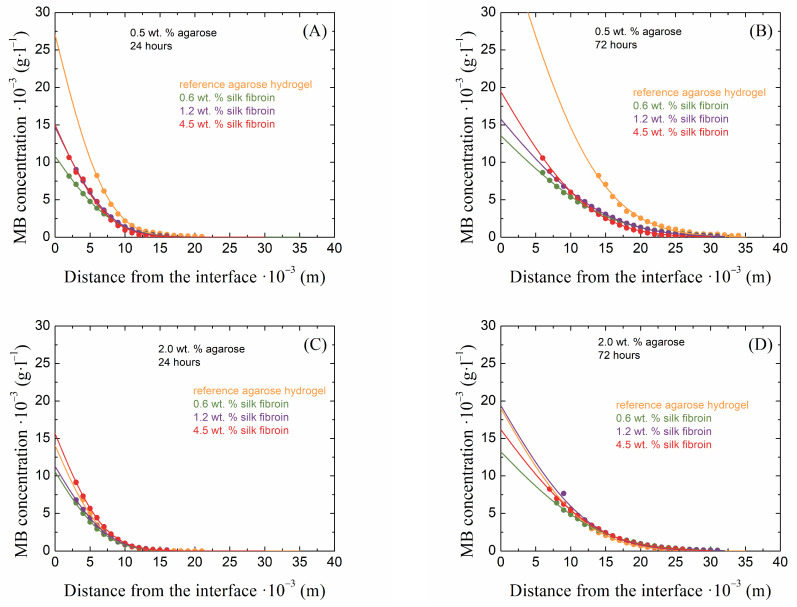
Examples of concentration profiles collected from 0.5 wt. % (**A**,**B**) and 2.0 wt. % (**C**,**D**) agarose hydrogels with the addition of silk fibroin at concentrations of 0.6, 1.2, and 4.5 wt. % after 24 (**A**,**C**) and 72 (**B**,**D**) hours of the diffusion of methylene blue.

The method of diffusion from a constant source is used to observe the penetration of a diffusion medium through a porous hydrogel material. How the presence of fibrous fibroin structures affects the transport properties of agarose hydrogels can be well described using this macroscopic observation. The principle of this continuous diffusion method is the spectrophotometric monitoring of the absorbance intensity of the diffusing dye, which permeates the hydrogel sample prepared in a cuvette after the sample is placed in a container with a solution of this dye for a certain time. Examples of the obtained concentration profiles of methylene blue are shown in Figure 5 for the lowest and highest concentrations of agarose and with silk fibroin additions after 24 and 72 h of diffusion.

**Figure 6:**
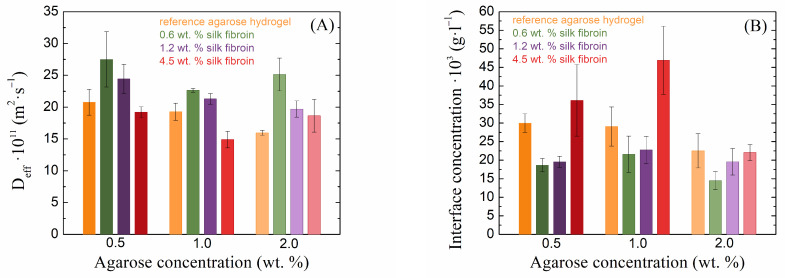
Values of effective diffusion coefficients (**A**) and theoretical concentrations at the hydrogel interface (**B**) of methylene blue for silk fibroin-modified agarose hydrogels.

The transport velocity of the diffusion probe is one of the most important characteristics in the case of hydrogel applications. For these applications, the diffusion must be controllable. In the case of the combination of agarose–fibroin hydrogels, there are different effects on the speed of transport of the diffusion probe inside these materials (Figure 6A). In the case of agarose hydrogels, as their concentration increased, the diffusion velocity of the diffusing dye slowed down, which is related to the crosslinking density of the hydrogel. In combination with silk fibroin, this transport can be both accelerated and slowed down. In our study, as the concentration of fibroin increased, the effective diffusion coefficient decreased regardless of the agarose concentration, but at the lower concentrations of fibroin (0.6 and 1.2 wt. %) in 0.5 and 1.0 wt. % agarose, it exceeded that in pure agarose hydrogels. In 2.0 wt. % agarose, fibroin at all concentrations increased the methylene blue diffusion coefficient. The decrease in the transport speed of the diffusion medium with increasing fibroin concentration occurs because vacancies in the agarose network are gradually filled with silk fibroin and also because the effect of the negatively charged protein is manifested; this prevents the positively charged dye from passing through the hydrogel. The increased effective diffusion coefficients in the case of less concentrated fibroin additions are again related to the rheologically determined mesh sizes.

Different concentrations of silk fibroin have very different effects on the barrier properties of the resulting hydrogels (Figure 6B). In the case of pure agarose, it can be observed that as its concentration increases, the theoretical concentration of the dye at the interface at the edge of the cuvette decreases slightly. This phenomenon is caused by the presence of a denser hydrogel network, which prevents the passage of a larger amount of dye. With the addition of silk fibroin at lower concentrations, this concentration was markedly reduced in all samples, which was also related to the mesh size and the fact that the presence of fibroin expanded the mesh size and allowed the dye to diffuse more effectively through the hydrogel. However, with a further increase in fibroin concentration, the interface concentration increased, and, in the case of the highest fibroin concentration, the concentration at the interface was even higher than that of the pure agarose gel. Since fibroin is a negatively charged protein and methylene blue is a positively charged dye, in this case, the amount of fibroin exceeded that of the agarose, resulting in the fibroin preventing the passage of methylene blue. At a high concentration of agarose, these barrier properties were not as perspicuous because the amount of agarose exceeded the amount of fibroin.

**Figure 7:**
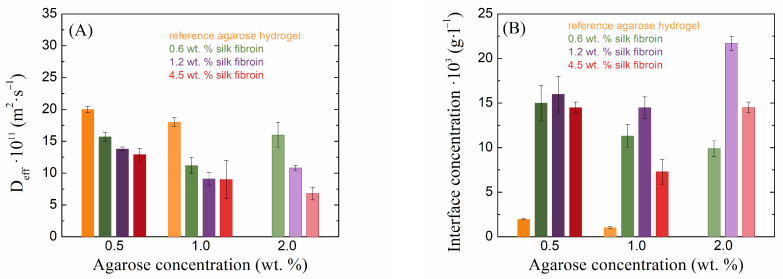
Values of effective diffusion coefficients (**A**) and theoretical concentrations at the hydrogel interface (**B**) of eosin-B for silk fibroin-modified agarose hydrogels.

A different behavior, with respect to transport properties, was shown by the second, oppositely (i.e., negatively) charged dye eosin-B (Figure 7). Unfortunately, in this case, it was not possible to determine the effective diffusion coefficient in the pure 2 wt. % agarose gel, because of the sample’s turbidity, through which it was not possible to follow the diffusion of eosin-B. The addition of silk fibroin decreased the diffusion coefficient of eosin-B and increased its concentration at the interface. Thus, in contrast to methylene blue, eosin-B showed faster transport in pure hydrogels (without the addition of silk fibroin) and “accumulated” much more at the interface of fibroin-added hydrogels than methylene blue. As the fibroin concentration increased, the diffusion coefficient of eosin-B decreased, while the effect of the fibroin concentration on the interface concentration showed no systematic trend. Silk fibroin thus demonstrated stronger barrier properties to eosin-B penetration than to methylene blue penetration. The main reason for this difference should be the electrostatic repulsion between the equally (negatively-)charged fibroin and eosin-B moieties. Interestingly, no effects of potential electrostatic binding between oppositely charged fibroin and methylene blue were observed, except perhaps the increased interface concentration for the highest fibroin concentration in 0.5 and 1.0 wt. % agarose (Figure 6).

The diffusion experiments in agarose–fibroin hydrogels showed that transport properties were influenced by both hydrogel mesh size and the charge of the diffusion probes. As expected, increasing the agarose concentration slowed the diffusion of methylene blue due to denser crosslinking. However, lower fibroin concentrations increased diffusion in less dense agarose gels by expanding the mesh, while higher fibroin concentrations reduced diffusion, likely due to fibroin filling network vacancies and interacting electrostatically with methylene blue. In contrast, the negatively charged eosin-B exhibited slower diffusion and greater accumulation at the interface, likely due to electrostatic repulsion with fibroin. These findings demonstrate that fibroin modulates diffusion behavior in a concentration-dependent manner and acts as a selective barrier, with stronger effects on negatively charged molecules, further confirming the relationship between fibroin’s impact on both the structure and transport properties of agarose hydrogels.

## 3. Conclusions

This work was focused on the influence of silk fibroin derived from B. Mori cocoons on physically crosslinked agarose hydrogels and their physicochemical properties (rheological and transport). It was intended as a simple model study of the effects of fibrous structures incorporated in a hydrogel on hydrogel properties.

This study demonstrated that the addition of silk fibroin to agarose hydrogels has a significant impact on their rheological and transport properties, depending on the concentration of both constituents. While the overall viscoelastic behavior remained unchanged, the specific rheological properties were modulated by the fibroin concentration. The influence on transport properties, with respect to the diffusion of two dyes differing in their charge, combined the same effects as presented for the rheological properties and the effect of electrostatic interactions between charged groups. The transport properties of diffusion probes in agarose–fibroin hydrogels were highly dependent on the concentrations of both agarose and silk fibroin. The barrier effect of fibroin was particularly evident for the negatively charged eosin-B due to electrostatic repulsion.

Overall, the findings illustrate the nuanced impact of fibroin supramolecular structures on agarose hydrogels, affecting their structure, mechanical properties, and transport behavior in a concentration-dependent manner. Silk fibroin, as a macromolecular fibrous structure introduced into the polysaccharide (agarose) matrix, modified the hydrogel properties through both physical and chemical interactions. By optimizing the ratio of silk fibroin to agarose, we demonstrated that mechanical and transport properties are closely linked, and the modification of one often affects the other. These results enhance our understanding of how supramolecular fibers influence the hydrogel network, contributing to insights into extracellular matrix behavior and supporting the development of tailored hydrogel materials crucial for potential biomedical applications.

## 4. Materials and Methods

### 4.1. Chemicals and Preparation of Materials

Agarose (Agarose E, Condalab, Madrid, Spain), methylene blue (Sigma-Aldrich, Prague, Czech Republic), and eosin-B (Sigma-Aldrich, Prague, Czech Republic) were used without further purification. The structural formulas and molecular weights of the dyes used are given in Supplementary Materials (Table S2 in Supplementary Materials).

Silk fibroin was extracted from raw silk fibers as per earlier established protocols [58]. Briefly, fibers were degummed for 30 min in 0.02 M sodium carbonate (Penta chemicals, Prague, Czech Republic) at 90 °C, thoroughly washed with distilled water, and dried at 60 °C overnight. The degummed fibers were dissolved in Ajisawa’s reagent consisting of calcium chloride (Lach-Ner, Neratovice, Czech Republic), water, and ethanol (Penta chemicals, Prague, Czech Republic) at a ratio of 1:8:2 for 6 h at 70 °C. The dissolved silk fibroin solution was then centrifuged (6000 rpm, 15 min) to separate undissolved fragments and impurities and dialyzed against deionized water using a 3.5 kDa molecular weight cutoff cellulose dialysis membrane for 3 days. The concentration of the obtained silk fibroin solution was determined from the dry matter weight as 8 wt. %, and it was stored at 4 °C for a maximum of 5 days for further studies.

The extracted silk fibroin solution was characterized by several techniques: infrared spectroscopy (FT-IR spectrometer NICOLET IS 50, Thermo Fisher Scientific, Waltham, MA, USA; ATR, 32 scans, resolution 4), dynamic light scattering (colloid analyzer ZETA NANO ZS, Malvern Instruments, Malvern, UK; 1 mL diluted sample, 25 °C) allowing zeta potential measurement, and differential scanning calorimetry (DSC analyzer Q2000, TA Instruments, TA Instruments, New Castel, DE, USA; aluminum pan, 2 °C/min).

### 4.2. Preparation of Hydrogels

Agarose hydrogels were prepared by means of the thermoreversible gelation of its aqueous solution. The procedure followed was consistent with our standard protocol [59]. An accurately weighed amount of agarose powder (concentration of agarose in hydrogel samples: 0.5 wt. %, 1 wt. %, and 2 wt. %) was added to deionized water and dissolved by slowly heating the solution in a water bath at 80 °C until the occurrence of a transparent solution. A tempered ultrasonic bath was used to degas the solution. Agarose–silk fibroin samples were prepared by adding a specific amount of silk fibroin solution (concentration of silk fibroin in hydrogel samples: 0.6 wt. %, 1.2 wt. %, and 4.5 wt. %) to a fully dissolved agarose solution before sonification. For the rheological experiments, samples were prepared in beakers, and for the diffusion experiments, samples were slowly poured into PMMA spectrometric cuvettes (inner dimension: 10 × 10 × 45 mm). The gentle cooling of samples covered by parafilm (to prevent drying) led to solidification and formation of the final hydrogels.

### 4.3. Rheological Characterization of Materials

As an appropriate technique for studying hydrogel mechanical properties, oscillatory rheology was performed using an Anton Paar MCR72 rotational rheometer employing cross-hatched 25 mm parallel plate geometry. The rheological procedure consists of an amplitude sweep test (with constant frequency oscillation) and a frequency sweep test (with constant amplitude of deformation). The amplitude sweep measurement is useful for determining the linear viscoelastic region (LVER, determined from the tolerance range of ± 5% deviation for G′ around the plateau value according to the standards ISO 6721-10 [60]) where the applied deformation is non-destructive, an important factor for the following frequency sweep test. In addition, this measurement also provides other important information about the behavior of the gel sample. The values of the viscoelastic moduli G′ and G″ provide information about the crosslinking density of the hydrogel network, and their crossover point corresponds to the transition between the solid-like and liquid-like behavior of the material [61,62]. Some of the structural parameters can be calculated from frequency sweep tests. Crosslinking density $\rho_{x}$ (mol·m^−3^) can be calculated using the value of the elastic modulus in the plateau $G_{e}$ (Pa), the universal gas constant R (J·mol^−1^·K^−1^), and the thermodynamic temperature T (K) [63,64,65]:

(1) $$\rho_{x}=\frac{G_{e}}{RT}$$

Then, with the assumption of spherical meshes in the network, the mesh size ξ (m) can be calculated using the crosslinking density and Avogadro’s number $N_{A}$ [66]:

(2) $$\xi =\sqrt[{3}]{\frac{6}{\pi \rho_{x}N_{A}}}$$

Both tests were carried out on freshly prepared samples in at least two repetitions (using freshly loaded samples for each). The standard deviations from the means are presented as error bars in the graphs or included in the table. Error bars are omitted from the graphs with rheometer data to maintain clarity. Before each measurement, a conditioning step was performed when the sample was loaded into the rheometer and left under the measurement conditions for 300 s. Both tests were performed at a temperature of 25 °C (preliminary experiments show minimal difference for results at 25 and 37 °C) with a 1000 µm measuring gap. Amplitude sweep tests were performed within the amplitude strain range of 0.01–1000% under a constant 1 Hz oscillation frequency, and frequency sweep tests were performed within the oscillation frequency range of 0.01–1000 rad·s^−1^ under a constant 0.1% amplitude strain chosen from LVER.

### 4.4. Diffusion Experiments

**Figure 8:**
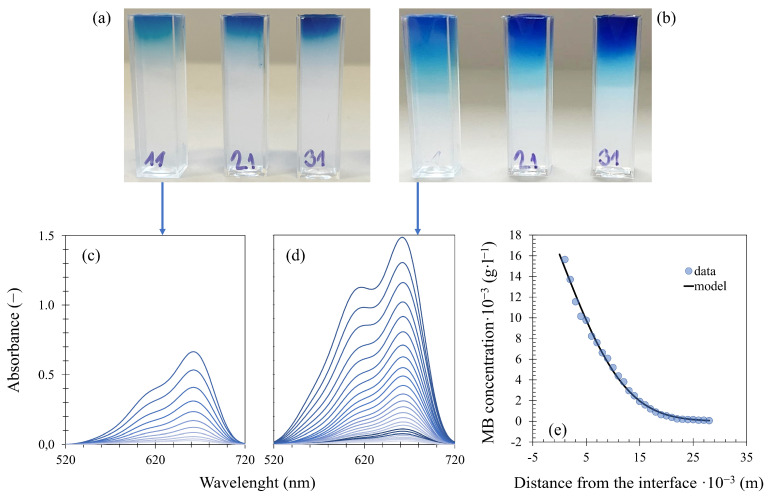
Experimental setup and analysis of diffusion from a constant source: hydrogel samples after 24 (**a**) and 72 (**b**) hours of methylene blue diffusion, collected spectra of selected (0.5 wt. % agarose with 0.6 wt. % fibroin) samples (**c**) and (**d**), and (**e**) concentration profile from (**d**) fitted by Equation (3).

Non-stationary diffusion experiments were performed to study the transport properties of silk fibroin-modified agarose hydrogels. In this macroscopic observation (graphically represented by Figure 8), hydrogel samples prepared in a cuvette are immersed in the dye solution, and after a defined time, UV-VIS spectra are collected at certain distances from the hydrogel/solution interface, which allows the creation of concentration profiles, i.e., the dependence of dye concentration on position in the hydrogel. A mathematical formula originating from Fick’s law can be used to fit each concentration profile and the effective diffusion coefficient $D_{eff}$ (m^2^·s^−1^) can be determined:

(3) $$c_{x}{=c}_{0}ERFC\frac{x}{\sqrt{4D_{eff}t}}$$

where $c_{x}$ and $c_{0}$ are the concentrations of dye in the hydrogel (g·L^−1^) at a distance x (m) from the hydrogel–solution interface or at the interface, respectively, and t is the time of diffusion [67,68].

The prepared hydrogel-filled cuvettes were immersed in a 0.01 g·L^−1^ aqueous solution of methylene blue and eosin-B with continuous stirring (via a magnetic stirrer at 250 RPM), and the dye was left to diffuse from the solution into the hydrogel samples. At defined time intervals (24, 48, and 72 h for methylene blue; 72 and 196 h for eosin-B), the cuvettes were taken out of the dye solution. UV–Vis spectra were collected in the 400–800 nm (methylene blue) and 300–700 nm (eosin-B) spectral range at various distances from the hydrogel/solution interface using a Varian Cary 50 UV–Vis spectrophotometer equipped with a special home-made accessory allowing spectra collection at different heights of the cuvette. The experiments were carried out at a temperature of 25 °C, each with at least two repetitions. The standard deviations from the means are presented as error bars in the graphs. The concentrations of both dyes in the samples were determined on the basis of calibration with a set of reference hydrogels with various known concentrations of methylene blue and eosin-B. The concentration profiles were fitted by Equation (3) using the Solver tool in MS Excel, giving the effective diffusion coefficient for every time interval, and the final value for each sample is the average of these individual coefficients.

## Data Availability

The data used in this study are available on request from the corresponding author.

## Supplementary Materials

The following supporting information can be downloaded at https://www.mdpi.com/article/10.3390/gels10100611/s1, Table S1: Values obtained from amplitude sweep rheology measurements for silk fibroin-modified agarose hydrogels; Table S2: Structural formulas and molecular weights of methylene blue and eosin-B; Figure S1: Amplitude sweep (A) and frequency sweep (B) tests of 1.0 wt. % agarose hydrogels with different concentrations of silk fibroin. Circles represent storage moduli G′ and triangles represent loss moduli G″; Figure S2: Amplitude sweep (A) and frequency sweep (B) tests of 2.0 wt. % agarose hydrogels with different concentrations of silk fibroin. Circles represent storage moduli G′ and triangles represent loss moduli G″.

## References

[1] Aswathy S.H., Narendrakumar U., Manjubala I. (2020). Commercial Hydrogels for Biomedical Applications. Heliyon.

[2] Ahmed E.M. (2015). Hydrogel: Preparation, Characterization, and Applications: A Review. J. Adv. Res..

[3] Bashir S., Hina M., Iqbal J., Rajpar A.H., Mujtaba M.A., Alghamdi N.A., Wageh S., Ramesh K., Ramesh S. (2020). Fundamental Concepts of Hydrogels: Synthesis, Properties, and Their Applications. Polymers.

[4] Gulrez S.K.H., Al-Assaf S., Phillips G.O., Carpi A. (2011). Hydrogels: Methods of Preparation, Characterisation and Applications. Progress in Molecular and Environmental Bioengineering.

[5] Wichterle O., LÍM D. (1960). Hydrophilic Gels for Biological Use. Nature.

[6] Caló E., Khutoryanskiy V.V. (2015). Biomedical Applications of Hydrogels: A Review of Patents and Commercial Products. Eur. Polym. J..

[7] Kashyap N., Kumar N., Kumar M.N.V.R. (2005). Hydrogels for Pharmaceutical and Biomedical Applications. Crit. Rev. Ther. Drug Carrier Syst..

[8] Hamidi M., Azadi A., Rafiei P. (2008). Hydrogel Nanoparticles in Drug Delivery. Adv. Drug Deliv. Rev..

[9] Geckil H., Xu F., Zhang X., Moon S., Demirci U. (2010). Engineering Hydrogels as Extracellular Matrix Mimics. Nanomedicine.

[10] Moreno-Arotzena O., Meier J., del Amo C., García-Aznar J. (2015). Characterization of Fibrin and Collagen Gels for Engineering Wound Healing Models. Materials.

[11] Migliaresi C., Motta A., Migliaresi C., Motta A. (2014). Scaffolds for Tissue Engineering: Biological Design, Materials, and Fabrication.

[12] Badylak S.F. (2007). The Extracellular Matrix as a Biologic Scaffold Material. Biomaterials.

[13] González-Díaz E., Varghese S. (2016). Hydrogels as Extracellular Matrix Analogs. Gels.

[14] Gattazzo F., Urciuolo A., Bonaldo P. (2014). Extracellular Matrix: A Dynamic Microenvironment for Stem Cell Niche. Biochim. Et Biophys. Acta (BBA)-Gen. Subj..

[15] Yue B. (2014). Biology of the Extracellular Matrix. J. Glaucoma.

[16] Frantz C., Stewart K.M., Weaver V.M. (2010). The Extracellular Matrix at a Glance. J. Cell Sci..

[17] Hynes R.O. (2009). The Extracellular Matrix: Not Just Pretty Fibrils. Science.

[18] Badylak S., Freytes D., Gilbert T. (2009). Extracellular Matrix as a Biological Scaffold Material: Structure and Function. Acta Biomater..

[19] Bhattacharyya S., Guillot S., Dabboue H., Tranchant J.-F., Salvetat J.-P. (2008). Carbon Nanotubes as Structural Nanofibers for Hyaluronic Acid Hydrogel Scaffolds. Biomacromolecules.

[20] Ma P.X., Zhang R. (1999). Synthetic Nano-Scale Fibrous Extracellular Matrix. J. Biomed. Mater. Res..

[21] Seal B. (2001). Polymeric Biomaterials for Tissue and Organ Regeneration. Mater. Sci. Eng. R Rep..

[22] Murphy N.P., Lampe K.J. (2015). Mimicking Biological Phenomena in Hydrogel-Based Biomaterials to Promote Dynamic Cellular Responses. J. Mater. Chem. B.

[23] de Moraes M.A., Paternotte E., Mantovani D., Beppu M.M. (2012). Mechanical and Biological Performances of New Scaffolds Made of Collagen Hydrogels and Fibroin Microfibers for Vascular Tissue Engineering. Macromol. Biosci..

[24] Aprodu A., Mantaj J., Raimi-Abraham B., Vllasaliu D. (2019). Evaluation of a Methylcellulose and Hyaluronic Acid Hydrogel as a Vehicle for Rectal Delivery of Biologics. Pharmaceutics.

[25] Perale G., Rossi F., Sundstrom E., Bacchiega S., Masi M., Forloni G., Veglianese P. (2011). Hydrogels in Spinal Cord Injury Repair Strategies. ACS Chem. Neurosci..

[26] Tibbitt M.W., Anseth K.S. (2009). Hydrogels as Extracellular Matrix Mimics for 3D Cell Culture. Biotechnol. Bioeng..

[27] Rowley J.A., Madlambayan G., Mooney D.J. (1999). Alginate Hydrogels as Synthetic Extracellular Matrix Materials. Biomaterials.

[28] Zarrintaj P., Manouchehri S., Ahmadi Z., Saeb M.R., Urbanska A.M., Kaplan D.L., Mozafari M. (2018). Agarose-Based Biomaterials for Tissue Engineering. Carbohydr. Polym..

[29] Dutta S.D., Patel D.K., Lim K.-T. (2019). Functional Cellulose-Based Hydrogels as Extracellular Matrices for Tissue Engineering. J. Biol. Eng..

[30] Arakawa C., Ng R., Tan S., Kim S., Wu B., Lee M. (2017). Photopolymerizable Chitosan-Collagen Hydrogels for Bone Tissue Engineering. J. Tissue Eng. Regen. Med..

[31] Oliveira J.T., Martins L., Picciochi R., Malafaya P.B., Sousa R.A., Neves N.M., Mano J.F., Reis R.L. (2010). Gellan Gum: A New Biomaterial for Cartilage Tissue Engineering Applications. J. Biomed. Mater. Res. A.

[32] Oliveira A.C.B., Morais T.F.L., Plepis A.M.G., Menezes P.F.C., Perussi J.R. (2015). Development of an in Vitro Dermis Equivalent Model Using Collagen-Based Scaffold. Photodiagn. Photodyn. Ther..

[33] Ren Y.-J., Zhou Z.-Y., Cui F.-Z., Wang Y., Zhao J.P., Xu Q.Y. (2009). Hyaluronic Acid/Polylysine Hydrogel as a Transfer System for Transplantation of Neural Stem Cells. J. Bioact. Compat. Polym..

[34] Neffe A.T., Julich-Gruner K.K., Lendlein A. (2014). Combinations of Biopolymers and Synthetic Polymers for Bone Regeneration. Biomaterials for Bone Regeneration.

[35] Renn D.W. (1984). Agar and Agarose: Indispensable Partners in Biotechnology. Ind. Eng. Chem. Prod. Res. Dev..

[36] Singh Y.P., Bhardwaj N., Mandal B.B. (2016). Potential of Agarose/Silk Fibroin Blended Hydrogel for in Vitro Cartilage Tissue Engineering. ACS Appl. Mater. Interfaces.

[37] Stephen A.M., Philips G.O., Williams P.A., Stephen A.M., Phillips G.O. (2006). Food Polysaccharides and Their Applications.

[38] Ramzi M., Rochas C., Guenet J.-M. (1998). Structure−Properties Relation for Agarose Thermoreversible Gels in Binary Solvents. Macromolecules.

[39] Normand V., Lootens D.L., Amici E., Plucknett K.P., Aymard P. (2000). New Insight into Agarose Gel Mechanical Properties. Biomacromolecules.

[40] Xu F., Dawson C., Lamb M., Mueller E., Stefanek E., Akbari M., Hoare T. (2022). Hydrogels for Tissue Engineering: Addressing Key Design Needs Toward Clinical Translation. Front. Bioeng. Biotechnol..

[41] Yun E.J., Yu S., Park N.J., Cho Y., Han N.R., Jin Y.-S., Kim K.H. (2021). Metabolic and Enzymatic Elucidation of Cooperative Degradation of Red Seaweed Agarose by Two Human Gut Bacteria. Sci. Rep..

[42] Irastorza-Lorenzo A., Sánchez-Porras D., Ortiz-Arrabal O., de Frutos M.J., Esteban E., Fernández J., Janer A., Campos A., Campos F., Alaminos M. (2021). Evaluation of Marine Agarose Biomaterials for Tissue Engineering Applications. Int. J. Mol. Sci..

[43] Mulinti P., Brooks J.E., Lervick B., Pullan J.E., Brooks A.E. (2018). Strategies to Improve the Hemocompatibility of Biodegradable Biomaterials. Hemocompatibility of Biomaterials for Clinical Applications.

[44] Ebrahimi A., Sadrjavadi K., Hajialyani M., Shokoohinia Y., Fattahi A. (2018). Preparation and Characterization of Silk Fibroin Hydrogel as Injectable Implants for Sustained Release of Risperidone. Drug Dev. Ind. Pharm..

[45] Zhang J., Allardyce B.J., Rajkhowa R., Kalita S., Dilley R.J., Wang X., Liu X. (2019). Silk Particles, Microfibres and Nanofibres: A Comparative Study of Their Functions in 3D Printing Hydrogel Scaffolds. Mater. Sci. Eng. C.

[46] Rockwood D.N., Preda R.C., Yücel T., Wang X., Lovett M.L., Kaplan D.L. (2011). Materials Fabrication from Bombyx Mori Silk Fibroin. Nat. Protoc..

[47] Lv Q., Hu K., Feng Q., Cui F. (2008). Fibroin/Collagen Hybrid Hydrogels with Crosslinking Method: Preparation, Properties, and Cytocompatibility. J. Biomed. Mater. Res. A.

[48] Zhou J., Cao C., Ma X., Lin J. (2010). Electrospinning of Silk Fibroin and Collagen for Vascular Tissue Engineering. Int. J. Biol. Macromol..

[49] Lu Y., Zhang S., Liu X., Ye S., Zhou X., Huang Q., Ren L. (2017). Silk/Agarose Scaffolds with Tunable Properties via SDS Assisted Rapid Gelation. RSC Adv..

[50] Tomasetti L., Breunig M. (2018). Preventing Obstructions of Nanosized Drug Delivery Systems by the Extracellular Matrix. Adv. Healthc. Mater..

[51] Zheng H., Zuo B. (2021). Functional Silk Fibroin Hydrogels: Preparation, Properties and Applications. J. Mater. Chem. B.

[52] Zhang H., Li L.-L., Dai F.-Y., Zhang H.-H., Ni B., Zhou W., Yang X., Wu Y.-Z. (2012). Preparation and Characterization of Silk Fibroin as a Biomaterial with Potential for Drug Delivery. J. Transl. Med..

[53] Giubertoni G., Caporaletti F., Roeters S.J., Chatterley A.S., Weidner T., Laity P., Holland C., Woutersen S. (2022). In Situ Identification of Secondary Structures in Unpurified Bombyx Mori Silk Fibrils Using Polarized Two-Dimensional Infrared Spectroscopy. Biomacromolecules.

[54] Boulet-Audet M., Vollrath F., Holland C. (2015). Identification and Classification of Silks Using Infrared Spectroscopy. J. Exp. Biol..

[55] Hu X., Kaplan D., Cebe P. (2007). Effect of Water on the Thermal Properties of Silk Fibroin. Thermochim. Acta.

[56] Hu X., Shmelev K., Sun L., Gil E.S., Park S.H., Cebe P., Kaplan D.L. (2011). Regulation of Silk Material Structure by Temperature-Controlled Water Vapor Annealing. Biomacromolecules.

[57] Larson R.G. (1999). The Structure and Rheology of Complex Fluids.

[58] AJISAWA A. (1998). Dissolution of Silk Fibroin with Calciumchloride/Ethanol Aqueous Solution Studies on the Dissolution of Silk Fibroin. (IX). J. Sericultural Sci. Jpn..

[59] Kadlec M., Smilek J., Pekař M. (2023). Dynamic mechanical analysis of agarose hydrogels and its relationship to shear oscillation. AIP Conference Proceedings.

[60] (2019). Plastics—Determination of Dynamic Mechanical Properties—Part 10: Dynamic Shear and Torsional Moduli.

[61] Mezger T.G. (2018). Applied Rheology: With Joe Flow on Rheology Road.

[62] Mezger T.G. (2011). The Rheology Handbook: For Users of Rotational and Oscillatory Rheometers.

[63] Thompson B.R., Zarket B.C., Lauten E.H., Amin S., Muthukrishnan S., Raghavan S.R. (2020). Liposomes Entrapped in Biopolymer Hydrogels Can Spontaneously Release into the External Solution. Langmuir.

[64] Rubinstein M., Colby R.H. (2003). Polymer Physics.

[65] Ali W., Gebert B., Altinpinar S., Mayer-Gall T., Ulbricht M., Gutmann J.S., Graf K. (2018). On the Potential of Using Dual-Function Hydrogels for Brackish Water Desalination. Polymers.

[66] Pescosolido L., Feruglio L., Farra R., Fiorentino S., Colombo I., Coviello T., Matricardi P., Hennink W.E., Vermonden T., Grassi M. (2012). Mesh Size Distribution Determination of Interpenetrating Polymer Network Hydrogels. Soft Matter..

[67] Hlaváč J. (1981). Základy Technologie Silikátů.

[68] Crank J. (1956). The Mathematics of Diffusion.

