# Supplementary material for: Effect of Silk Fibroin on the Mechanical and Transport Properties of Agarose Hydrogels
Source: Gels. 2024 Sep 24;10(10):611. doi: 10.3390/gels10100611 (PMC11508024; doi:10.3390/gels10100611)
Supplement: Supplementary file 1 [file gels-10-00611-s001.zip › gels-3187277-supplementary.pdf]

Supplementary materials

Figure S1 Amplitude sweep (A) and frequency sweep (B) tests of 1.0 wt. % agarose hydrogels with different concentrations of silk fibroin. Circles represent storage moduli G' and triangles represent loss moduli G".

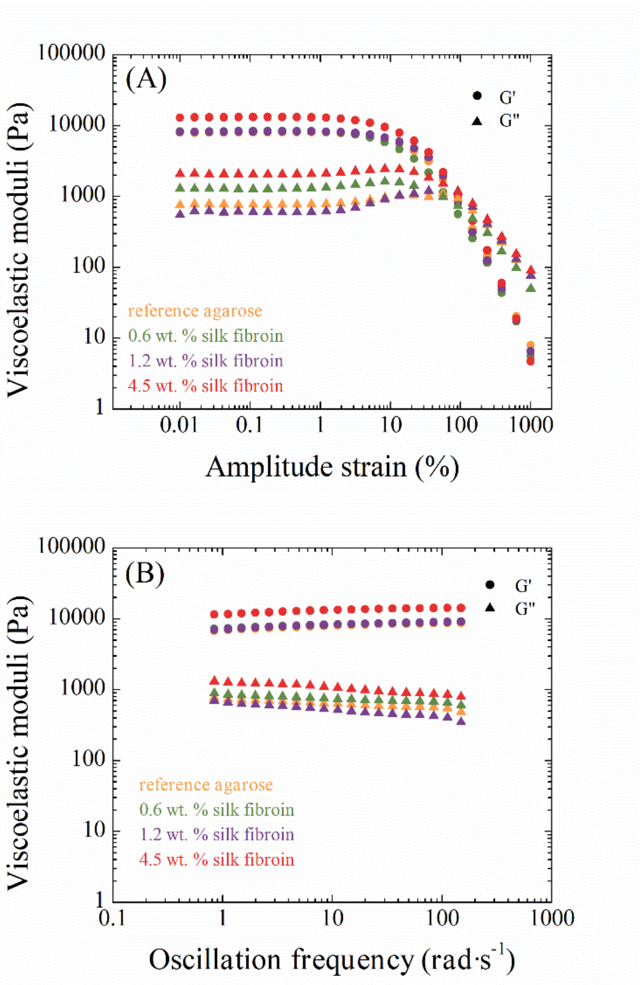

Figure S2 Amplitude sweep (A) and frequency sweep (B) tests of 2.0 wt. % agarose hydrogels with different concentration of silk fibroin. Circles represent storage moduli G' and triangles represent loss moduli G".

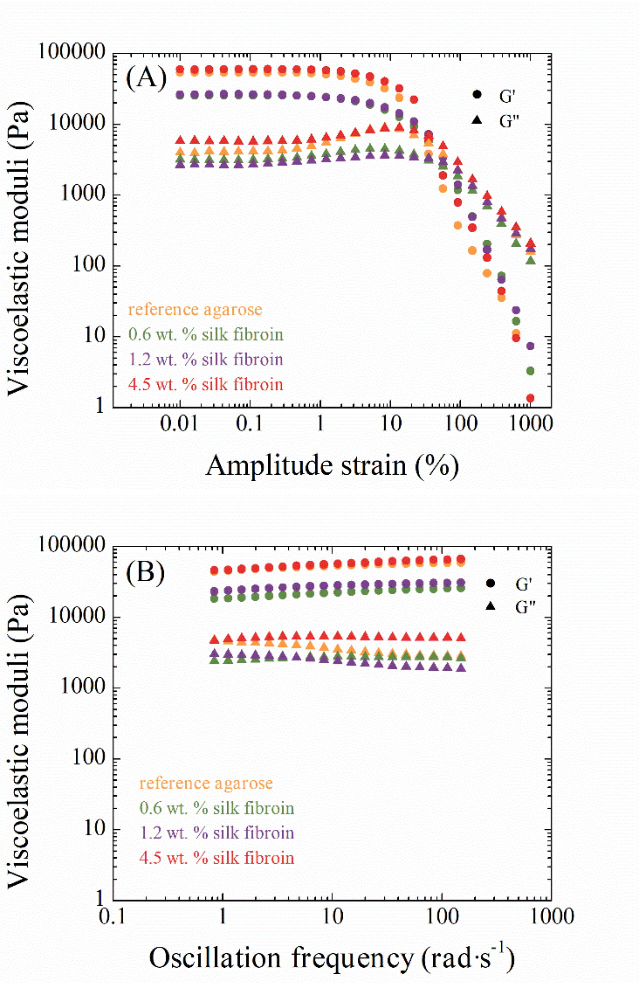

Table S1 Values obtained from rheology measurements for silk fibroin-modified agarose hydrogels.

Sample composition		*LVR end*	*Average moduli in LVR*		*Crossover point*		*Mesh size*
**Agarose** (wt. %)	**Fibroin** (wt. %)	**Strain** (%)	**G'** (Pa)	**G''** (Pa)	**G'** (Pa)	**Strain** (%)	**ξ** (nm)
0.5	×	5.13 ± 0.03	2913 ± 39	290 ± 10	365 ± 16	94 ± 7	13,95 ± 0.05
	0.6	2.56 ± 0.70	1796 ± 24	145 ± 12	224 ± 7	117 ± 42	17.00 ± 1.00
	1.2	3.16 ± 0.01	1903 ± 23	123 ± 6	316 ± 46	124 ± 31	16.20 ± 0.50
	4.5	1.59 ± 0.44	5503 ± 38	480 ± 17	680 ± 323	81 ± 7	12.00 ± 0.01
1	×	3.17 ± 0.01	7968 ± 122	777 ± 21	1086 ± 280	74 ± 5	9.96 ± 0.06
	0.6	1.96 ± 0.01	8280 ± 121	1297 ± 36	884 ± 15	70 ± 18	9.80 ± 0.30
	1.2	2.57 ± 0.70	8216 ± 72	605 ± 22	1352 ± 236	76 ± 4	9.84 ± 0.09
	4.5	1.96 ± 0.01	13051 ± 191	2076 ± 36	1233 ± 26	85 ± 6	8.41 ± 0.04
2	×	1.30 ± 0.27	53782 ± 473	4242 ± 294	6652 ± 278	27 ± 8	5.29 ± 0.01
	0.6	1.59 ± 0.43	25552 ± 261	3231 ± 146	2573 ± 556	62 ± 26	7.07 ± 0.02
	1.2	0.75 ± 0.01	26458 ± 84	2701 ± 47	3066 ± 152	63 ± 33	6.60 ± 0.20
	4.5	1.59 ± 0.44	59460 ± 642	5899 ± 213	7009 ± 91	33.3 ± 0.2	5.18 ± 0.05

Table S2 Structural formulas and molecular weights of methylene blue and eosin-B

Methylene blue	*Eosin B*
M = 319.85 g·mol^{-1}	M = 580.09 g·mol^{-1}
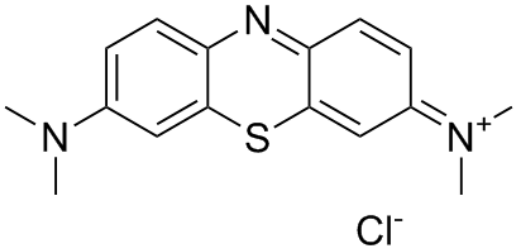	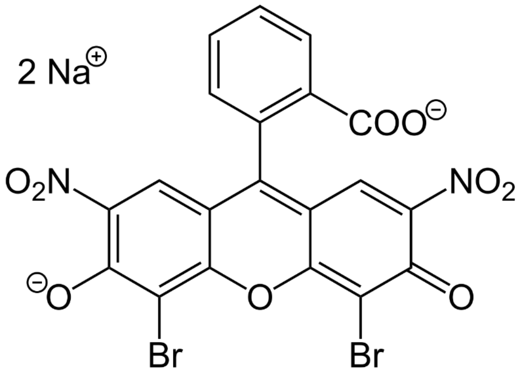
